# To see or not to see: does video CPR perform better than telephone CPR?

**DOI:** 10.1186/cc13677

**Published:** 2014-03-17

**Authors:** A Ghuysen, A Delfosse, S Stipulante, A Donneau, V D'Orio

**Affiliations:** 1 CHU - Ulg Liege, Belgium; 2 Federal Public Health Services, Liège, Belgium; 3 Liège University, Liege, Belgium

## Introduction

The ALERT algorithm, a simple and effective compression-only telephone CPR (t-CPR) protocol, has been previously demonstrated to help bystanders initiate CPR. According to their worldwide availability, mobile phone communications may play an increasing role in emergency calls. Preliminary studies suggest that they might improve dispatcher's understanding of the rescuer's situation. However, there is currently no validated protocol for videoconference-assisted CPR (v-CPR). We initiated the present study to validate an original protocol of v-CPR based on the ALERT algorithm and to evaluate the potential benefit of this assistance in comparison with classical t-CPR.

## Methods

We developed a strictly worded algorithm for v-CPR, adapted from the ALERT t-CPR protocol, with additional re-evaluation loops, every 2 minutes. A total of 120 students without prior CPR training were recruited from upper secondary school, during regular class hours, and randomly assigned to the t-CPR group (*n *= 60) versus the v-CPR group (*n *= 60). The Resusci^®^Anne SkillReporter™ manikin was used to evaluate CPR performance. Data were transferred from the manikin into a computerized database using the Laerdal SkillReporting System V.2.2.1 software. Further analysis was based on audio-recordings and video-recordings. Primary outcome measures were the results of the Cardiff evaluation test; the secondary measures were global scoring of a complete 7-minute period of CPR.

## Results

The mean chest compression rate increased significantly in the v-CPR group as compared with t-CPR (110 ± 16 vs. 86 ± 28; *P *< 0.0001), while depth remained constant (48 ± 13 mm vs. 47 ± 16 mm, *P = *NS). Hand positioning was correct in 91.7% of cases with v-CPR, but only in 68% with t-CPR (*P *= 0.001). The hands-off period was almost nonexistent in the v-CPR group (0 vs. 7 seconds; *P *= 0.0016), but the median no-flow time was significantly greater in the v-CPR group (146 vs. 122 seconds, *P <*0.0001). As a consequence, global evaluation of CPR performance revealed a significant improvement in v-CPR group score as compared with the t-CPR group (6 vs. 5, *P <*0.001).

## Conclusion

Video-assisted CPR using this original algorithm allows bystanders to reach compression rates and depths close to international guidelines and to reduce hands-off events during CPR.

